# Direct and real-time observation of hole transport dynamics in anatase TiO_2_ using X-ray free-electron laser

**DOI:** 10.1038/s41467-022-30336-1

**Published:** 2022-05-09

**Authors:** Sang Han Park, Abhishek Katoch, Keun Hwa Chae, Sanjeev Gautam, Piter Miedema, Sang Wan Cho, Minseok Kim, Ru-Pan Wang, Masoud Lazemi, Frank de Groot, Soonnam Kwon

**Affiliations:** 1 Pohang Accelerator Laboratory, Pohang, Gyeongbuk 37673 South Korea; 2 Korea Institute of Science and Technology, Seoul, South Korea; 3 Dr S. S. Bhatnagar University Institute of Chemical Engineering & Technology, Panjab University, Chandigarh, 160014 India; 4 European XFEL Gmbh, Notkestrasse 85, D-22607 Hamburg, Germany; 5 Department of Physics, Yonsei Univerity, Wonju, South Korea; 6 Materials Chemistry and Catalysis (MCC), Debye Institute for Nanomaterials Science, Utrecht University, Universiteitslaan 99, 3584 CG Utrecht, The Netherlands; 7 Department of Physics, University of Hamburg, Luruper Chaussee 149, G610, 22761 Hamburg, Germany

**Keywords:** Ultrafast photonics, Photocatalysis

## Abstract

Carrier dynamics affects photocatalytic systems, but direct and real-time observations in an element-specific and energy-level-specific manner are challenging. In this study, we demonstrate that the dynamics of photo-generated holes in metal oxides can be directly probed by using femtosecond X-ray absorption spectroscopy at an X-ray free-electron laser. We identify the energy level and life time of holes with a long life time (230 pico-seconds) in nano-crystal materials. We also observe that trapped holes show an energy distribution in the bandgap region with a formation time of 0.3 pico-seconds and a decay time of 8.0 pico-seconds at room temperature. We corroborate the dynamics of the electrons by using X-ray absorption spectroscopy at the metal L-edges in a consistent explanation with that of the holes.

## Abstract (web summary)

Direct and real-time observation of the excess holes in an element-specific and energylevel-specific manner is a challenging task. Here the authors present a complete hole dynamics of photo-excited anatase TiO_2_ with 100 femto-second resolution.

## Introduction

Transition metal oxides (TMOs) are among the most important materials for photocatalysis due to their exceptional properties^1,2^. Understanding the carrier dynamics on TMOs is key to discovering ways to increase their catalytic activity. Titanium dioxide (TiO_2_) is one of the most-investigated TMOs with an empty 3d band^3^. Defects, polarons or adsorbates can lead to excess carriers that affect the critical properties of the catalyst^4–10^.

**Fig. 1:**
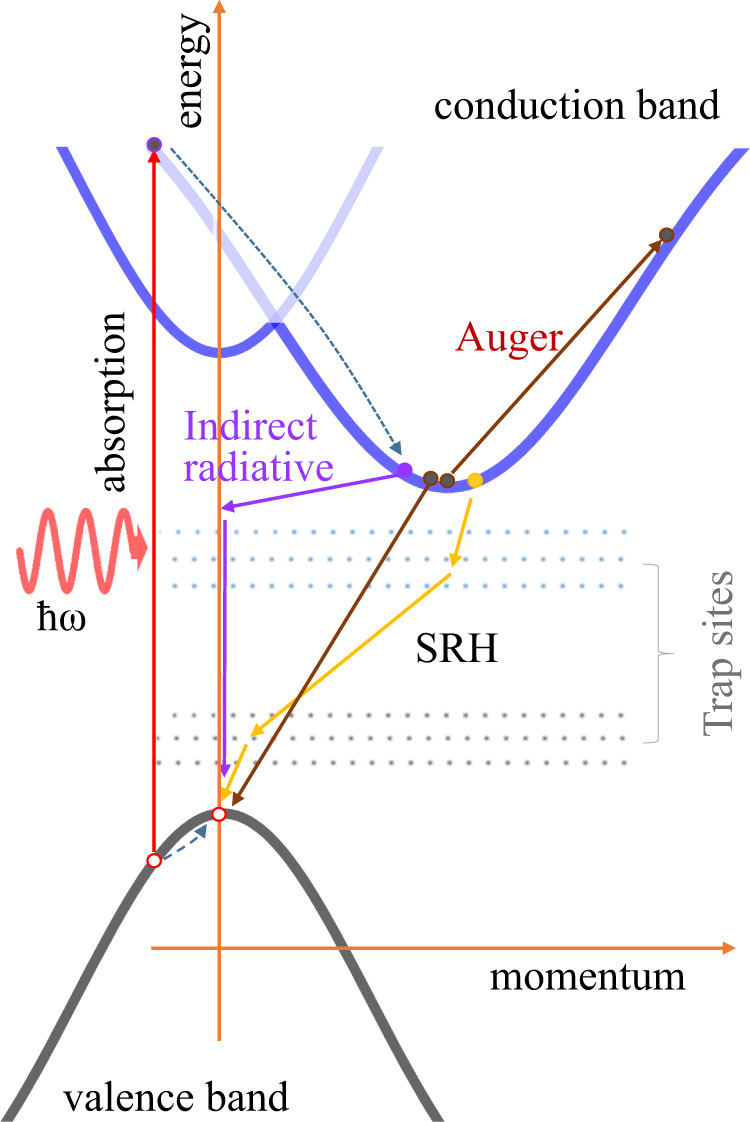
Schematic band diagram of anatase TiO_2_ which has an indirect band gap. The simplified optical excitation and possible decay processes are represented.

Photo-excitation creates free, and trapped carriers, or bound electron-hole pairs (excitons) with different kinetics (Fig. 1). When electrons absorb photons which have larger energy than the band gap, it creates highly energetic (hot) carriers well above conduction band minimum. The hot carriers thermalize into a Fermi–Dirac distribution with T_e_ or T_h_ < 0.05 ps and they reach thermal equilibrium with the lattice after ~0.5 ps through emission of longitudinal optical phonons^11^. Subsequently the hot carriers recombine through three mechanisms, respectively indirect radiative recombination through phonon assisted processes^12^, Auger recombination^13,14^, and defect assisted recombination known as Shockley–Read–Hall (SRH) recombination^15^.

A number of methods have been used to explore the dynamics of photo-excited electrons or holes in TiO_2_: Laser spectroscopy only detects indirect effects such as changes induced by carrier scavengers; it does not give any element or energy specific information (direct evidence) for electron or hole dynamics^2,3,16^. Electron paramagnetic resonance (EPR) can detect direct signals only when specific atoms (e.g. O^−^ or Ti^3+^) maintain excess charge; the method usually requires low temperatures, with the complication that the characteristics at low temperatures differ from those at room temperature^17,18^. Low-temperature scanning tunneling microscopy and spectroscopy can also measure excess carriers directly^19^. However, no direct observation of these processes in real-time was available before the development of pump-probe techniques that use X-rays. Hard X-ray pump-probe techniques obtain mixtures of electronic and structural information^20–22^. Time-resolved X-ray absorption spectroscopy (tr-XAS) at the Ti K-edge have demonstrated that electron localization at Ti atoms occurs in <100 fs to form Ti^3+^ centers, whereas the structural changes that complete the formation of a polaron require ~300 fs^21,22^. The excited electrons have two lifetime components, one of 0.3 ns and one of 6 ns^20^. However, direct evidence for hole dynamics has never been observed in TMOs, although indirect evidence has been reported for hole polarons in ZnO^23,24^ and in perovskites^25,26^.

Here, using tr-XAS of the oxygen K-edge and the Ti L_2,3_-edge, we investigated the dynamics of hole and electron in two different forms of anatase TiO_2_, single crystal (SC) and nano-crystal (NC). Our approach uses the oxygen K-edge XAS, which effectively probes the oxygen 2p projected density of states (pDOS) of the substance to investigate its hole dynamics^27–29^. By using a 266 nm optical pump with a fluence between 10 and 30 mJ/cm^2^, we could probe the carrier dynamics with a time resolution of ~120 ± 10 fs. Tr-XAS of the oxygen K-edge provides time-resolved information on the relative position of the energy of the carrier. For example, we can distinguish a hole in the valence band maximum (VBM) or 0.7 eV above the VBM, which has not been accessible with other methods. The results obtained provide the hole dynamics from its generation to transfer, trapping and recombination in an energy-position-specific manner. Our results agree with previous reports on the electron polaron (Ti^3+^) formation time (<0.7 ps) and the decay time constants (10~200 ps and >500 ps).

## Results

### Definitions of the observables found in the XAS spectra

**Fig. 2:**
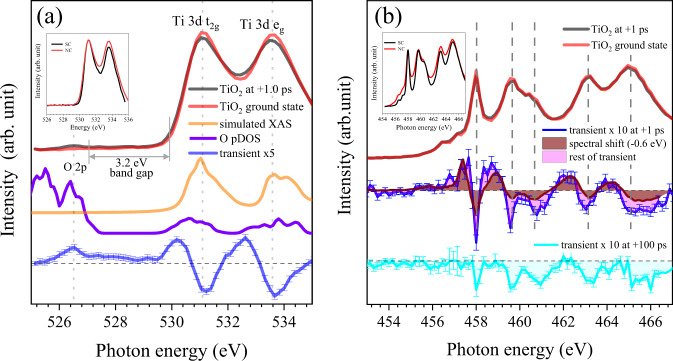
XAS spectra of 20 nm TiO_2_ NC at the oxygen K-edge and the Ti L-edge. **a** Overview of the oxygen K-edge XAS measured +1 ps after the pump excitation (black) and the ground states before excitation (red). Simulated XAS (orange) and O pDOS (green) are well matched with measured XAS at the oxygen K-edge. The transient XAS spectrum at +1 ps is multiplied by 5 times. (blue) (inset) ground state XAS spectra for SC and NC at the oxygen K-edge. **b** Overview of the Ti L-edge XAS measured +1 ps after the pump excitation (black) and the ground states before excitation (red) for anatase TiO_2_ NC. The transient XAS spectrum at +1 ps (blue) consists shift-induced component (green) and the other component (pink). The transient XAS spectrum at +100 ps (cyan) does not show shift induced component. (inset) ground state XAS spectra for SC and NC at the Ti L-edge. Error bars correspond to the standard errors.

The oxygen K-edge XAS spectra of a 20-nm anatase TiO_2_ NC were obtained before and after laser illumination. (Fig. 2a) The transient XAS spectra were obtained by subtracting a negative-delay (−15 ps) XAS spectrum of the ground state (GS) from a positive-delay XAS spectrum of the optically excited state. The XAS spectra show two peaks at 531.0 and 533.5 eV that represent the O 2p projected density of empty states due to metal–oxygen orbital mixing^27^. The XAS spectrum of the ground state can be simulated by using OCEAN package^30,31^. After laser excitation, a new peak at ~526.5 eV is seen in XAS, which implies that valence band holes were generated. The XAS features after laser excitation match the O 2p orbital pDOS obtained from an ab-initio calculation^32–34^.

The XAS spectra of anatase TiO_2_ NC, were also collected at Ti L_2, 3_-edges. (Fig. 2b) For both of the ground and excited state spectra, five well-split peaks at 458.0, 459.6, 460.7, 463.1, and 465.0 eV can be assigned to be Ti^4+^ in comparison with the previously-reported anatase TiO_2_ XAS spectra simulated by multi-channel multiple scattering theory method^35^. The transients at energies of Ti^4+^ peak-positions show negative changes. Whereas the transients at energies in between the Ti^4+^ peaks show positive changes. We assume this transient spectrum as a combination of rigid shift of the whole spectrum due to chemical shift caused by localized electron in Ti^3+^ site and suppression of Ti^4+^ due to the existence of extra electrons above VB. A detailed discussion will be given in the Ti L-edge section.

The insets of Fig. 2 show the comparison between SC and NC. The spectra of NC show broader spectral shape than that of SC, especially at the Ti L-edge. The larger surface component of NC can be visible in XAS spectra for a particle <20 nm^36,37^. The different amount of surface component will help to analyze the different nature of SC and NC.

### Carrier dynamics observed at the oxygen K-edge

**Fig. 3:**
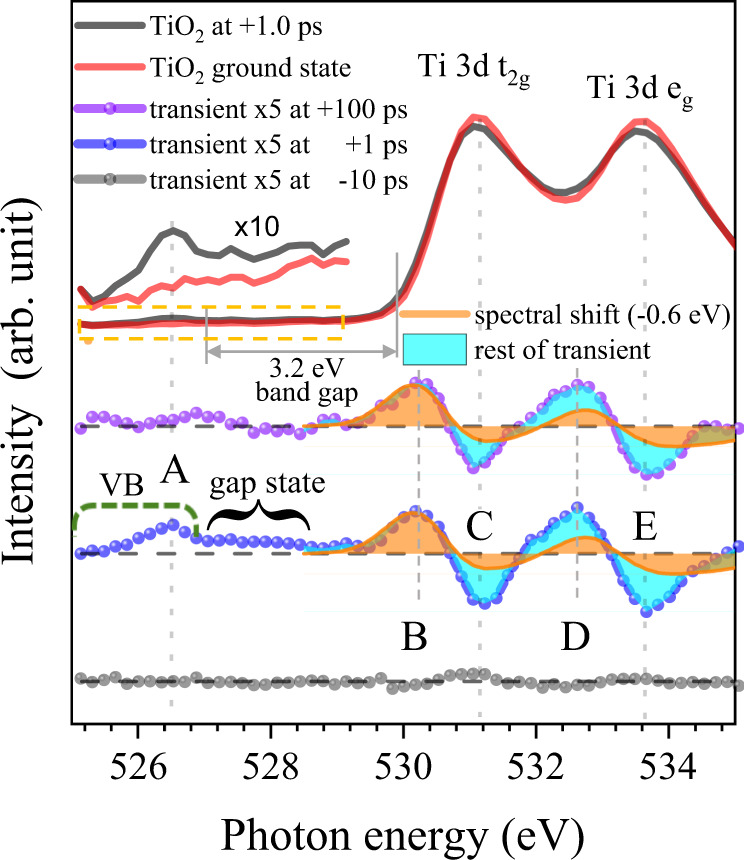
Femtosecond transient-absorption spectroscopy of TiO_2_ NC at the oxygen K-edge. XAS spectra measured +1 ps after pump excitation (black) and ground states before excitation (red). Transient XAS are obtained at +1 and +100 ps time delays. The transient XAS was obtained from the difference between the optically excited XAS at several pump-probe time delays and XAS measured at a negative time delay of −15 ps (ground state). Errors for the transient XAS were estimated from the transient XAS at a negative delay time (−10 ps).

The XAS spectra at the oxygen K-edge before and after laser illumination and the transient XAS spectra are shown in Fig. 3. Transient XAS spectra were recorded at 1, 100, and −10 ps with a 266 nm optical pump laser; apparently significant features in the transients are indicated as A–E. Feature A and the gap state show relatively fast decay within 100 ps, whereas the other features of B-E remain almost unchanged until 100 ps. Peak A and the indicated gap states (Fig. 3) can be assigned to holes in the oxygen 2p valence band (VB) and localized defect sites, respectively. The features B-E result from energy shifts of the excited states with respect to the ground state, which is evident from the subtraction of ground state spectrum from the shift (by −0.6 eV) of the whole spectrum. (Detailed simulation results can be found in supplementary information and Supplementary Fig. S8.) The optical excitation moves an electron from the oxygen VB to the conduction band (CB), so the charge density decreases at oxygen sites and increases at titanium sites. This transition will shift the Ti 3d bands and pDOS to lower energy, as observed.

**Fig. 4:**
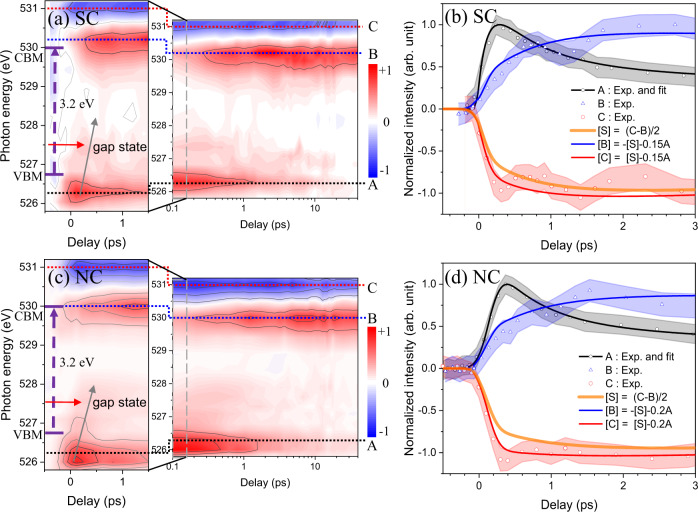
Initial behavior of photo-generated holes near the band gap region probed by oxygen K-edge. Experimental data of the transient signal as a function of pump-probe delay in the energy regions between VB and CB (525.8~531.2 eV) for anatase TiO_2_ (**a**) SC and (**c**) NC. Red: increased absorption intensity; blue: decreased absorption intensity; purple arrows: abrupt changes in transient signals by electron excitation; green arrows: traces of hole peak times from A to gap state. Red arrows: positions of center of gap states. The VBM and CBM were determined from XAS spectrum at oxygen K-edge by the linear extrapolation method. (Supplementary Fig. S2) **b**, **d** Normalized transient signals at three different photon energies (A (526.3 eV), B (530.2 eV for SC and 530.0 eV for NC), and C (531.0 eV).) during initial time delays for (**b**) SC and (**d**) NC. Experimental data (dot) agree well with calculation of our model (line). Shaded regions correspond to the standard errors.

The intensity variations of the transient signals in the energy region from 525.8 eV to 531.2 eV are shown in Fig. 4 for SC and NC. After the first appearance of holes (created by the pump laser), the transient maps (Fig. 4a, c) show that holes are moved from the valence band to defect states in the energy gap region with a certain time delay depending on the energy level. Gap states are located between 526.8 and 528.0 eV with a center at ~527.5 eV, which is ~0.7 eV above the VBM (526.8 eV). Features B-E (Fig. 3) can be assigned to an overall shift of the empty states as explained above, even though the transient spectrum cannot be perfectly reproduced only with overall shift as shown in Supplementary Information and Supplementary Fig. S8.

However, there still exist an unexplainable phenomena: in Fig. 4, feature B shows slower growth compared to the other feature C-E (if B-E are composed of only the rigid shift, they should show the same dynamics). We developed a model to explain this behavior. Peak A corresponds to hole creation and decay, and we represent this by curve A (*A*). Peak B is a combination of electron-hole (e-h) recombination and a shift. Peak C is a combination of the e-h recombination and an opposite-shift. Therefore, the transients of the unoccupied energy region (i.e., >530 eV) can be expressed as a combination of^1^ a shift function *S* (a negative shift transient signal with slow decay in Fig. 4b, d) and^2^ a function with fast-rising followed by fast decay (*αA*, where *α* represents a fitting parameter that varies with energy, and *A* is curve A). Function *S* is a result of the overall lower energy shift of the DOS of the excited state from that of the ground state as shown in Fig. 3, and function *αA* represents fast dynamics influenced by the recombination. We can formulate the transients of each peak (A, B, and C in Fig. 4) as

1 $$A\,{{{{{\rm{is}}}}}}\;{{{{{\rm{curve}}}}}}\;{{{{{\rm{A}}}}}}$$

2 $$S\,=(C-B)/2$$

3 $$B=-S-\alpha A$$

4 $$C=S-\alpha A$$

A model that uses the two functions (*A* and *S*) was compared to the experimental data (Fig. 4b, d) and reproduced all transients very well. We interpret this analysis as follows: *S* represents a shift of the whole range of empty states to lower energy due to the presence of extra electrons that do not show fast recombination and *αA* represents the electrons that do participate in the recombination with the holes (i.e. bound excitons).

**Fig. 5:**
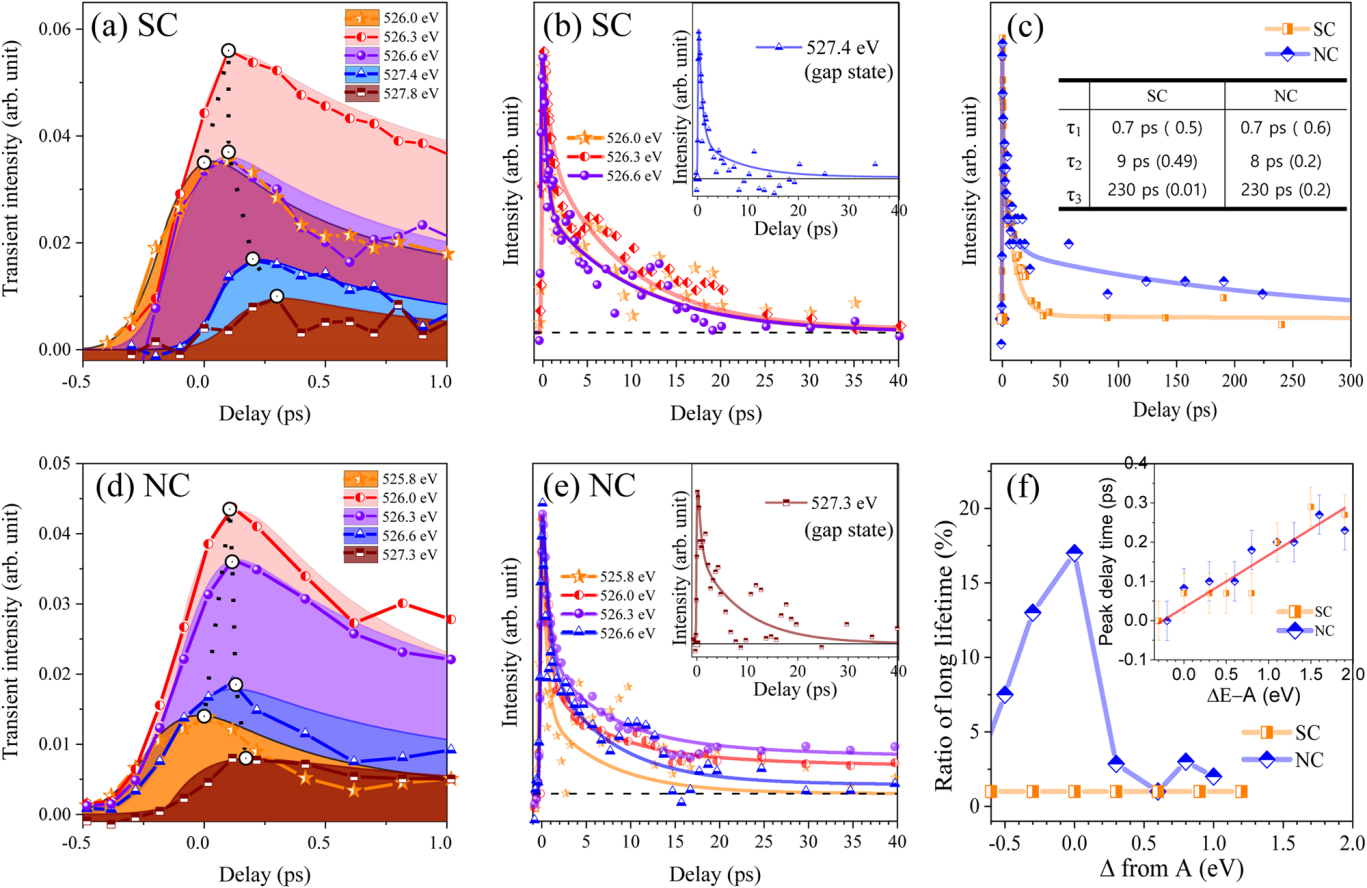
Behavior of photo-excited holes near A (526.3 eV) at extended times. Initial behaviors of hole transients at energy levels from below to above A for (**a**) SC and (**d**) NC. **b**, **e** Normalized kinetic trace of transient XAS around photon energies indicated as A. Insets: Transients at gap energies. In a SC (**b**), longer-lifetime components are negligibly small (**c**). In a NC (**e**), longer-lifetime components are observed as elevated baselines (**c** and **f**). The elevated baselines do not cross zero value at A. This corresponds to ~20 % at a time delay of 40 ps. The data are fitted using three exponential decay functions. **c** The hole transients at A for the delay time up to 300 ps. Inset: Decay time constant (amplitude) used to fit the three exponential decay functions. **f** Ratios of long lifetime (230 ps) component as a function of energy positions. Inset: Peak position vs energy position in **a** and **d**. Error bars correspond to the standard error.

XAS transients for holes at VB and the gap state are shown at extended times (Fig. 5). In the NC, near the VBM, the long-lifetime (230 ps) component has a larger amplitude than any other energies (Fig. 5 c, e, f). The transient of A can be fitted using three exponential decay functions that have lifetimes of 0.7, 8.0, and 230 ps, and an amplitude ratio of 0.6:0.2:0.2 (Fig. 5c). In a SC, the decay lifetimes are 0.7, 9.0, and 230 ps and the amplitude ratio is 0.5:0.49:0.01 (Fig. 5c). The short-lifetime components in the NC were similar to those in SC (0.7 and 8~9 ps). Whereas the long life time component is clearly seen only in NC.

Gap states are located in the region with photon energies of 526.8~528.5 eV. Gap states that can trap holes are one or more of the following: Ti interstitials (Ti_int_), oxygen vacancies (V_O_), or self-trapped hole states (or hole polarons)^6,9,38^. Gap states have shorter lifetimes than that of A of the NC (Fig. 4a, c)^39^. The initial decay is very fast and the long-time component is absent (insets in Fig. 5b, e). The gap states are observed without difference for both of SC and NC. For a SC, the lifetimes did not show obvious differences between transients near A and the other transients (Fig. 5b). In contrast, in the NC, the transients of the gap states had shorter lifetimes than the transients near A (Fig. 5e). The delay of the peak increased with increase in the energy position of holes with respect to A (Fig. 5f, inset). During the first time step of 0.1 ps, the peak times of hot holes (generated deep in the valence band) approaches to that of A. Our time resolution could be determined as 0.12 ± 0.01 ps from the fitting of time profiles using Heaviside function. The time scale of hot carrier cooling is shorter than our time resolution and consistent with previously reported results of optical transient absorption experiment^11^. The peak times of gap holes were observed from 0 to 0.3 ps.

### Carrier dynamics observed at the Ti L-edge

**Fig. 6:**
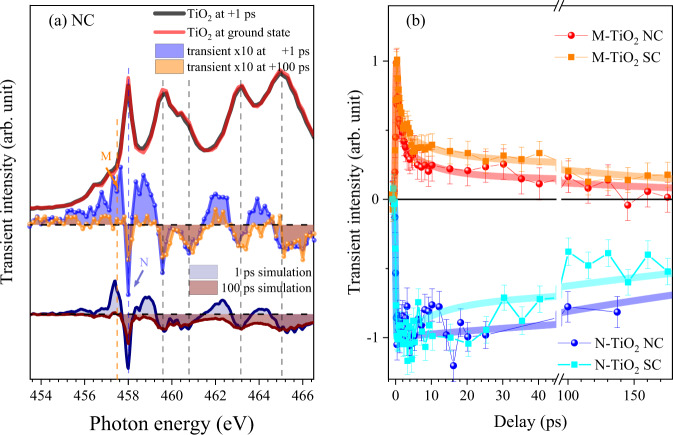
Transient-absorption spectroscopy of TiO_2_ at the Ti L_2_, 3-edge. **a** XAS measured at the excited and ground states for anatase TiO_2_ NC and transient XAS measured at a time delay of +1 and +100 ps. Simulated transient spectra are shown in comparison. (Supplementary Fig. S9). **b** Normalized transient curves at the photon energies marked as M and N in **a** as a function of time delay. All transient signals showed abrupt changes by optical pumping. The decays are much faster at M than at N. These behaviors together with those in **a** indicate that the positive changes are faster than negative changes. Error bars correspond to the standard error.

The XAS spectra at the Ti L-edge and the transient XAS spectra magnified by a factor of ten are shown in Fig. 6a which were measured at two different time delays. The transient of spectrum at 1 ps resembles that of subtraction of ground state spectrum from the shifted (−0.6 eV) spectrum. The shift of the spectrum can be assigned as chemical shift due to electron transfer to Ti atomic sites. This shift-induced component constitutes the majority of the transient at 1 ps. The remaining minor component at 1 ps is supposed to be caused by the broadening effect (resulting in the decrease of the peaks of ground state spectrum (Ti^4+^)) as shown in Supplementary Fig. S9b and Supplementary Eqs. (S3)–(5). The shift-induced transient XAS can be assigned as polaronic Ti^3+^or extra electrons in CB^40,41^. This transient XAS can be interpreted to be caused by the electron transfer from the O atom to the Ti atom, and a consequent decrease of Ti^4+^ and an increase of Ti^3+^. After 100 ps, the positive transient signal has almost disappeared, but a significant negative transient signal remains (Fig. 6a). The shift-induced components seem to almost disappear and only the decrease in Ti^4+^ peaks remains as shown in Supplementary Fig. S9c (we will call this as 4+ decrease hereafter). The time profiles at the two different energy positions were measured: M (457.5 eV) and N (458 eV), respectively. The transients that correspond to the peak positions of Ti^4+^ (N) showed an immediate decrease after excitation and retained the decreased intensity for >500 ps (Fig. 6b lower panel); i.e., the system does not recover to the original state in 500 ps. Whereas the transients of M increased immediately and decayed in 230 ps (Fig. 6b upper panel). For all samples of SC and NC, the decay curve can be fitted using two fast (0.7 and 8 ps) components and a slow (230 ps) component with an amplitude ratio of 0.6:0.2:0.2 (Fig. 6b upper panel). Most of the transients (80 %) disappeared in 8 ps and only 20 % survived to 230 ps. The physical meaning of these phenomena will be treated in Discussion section and Supplementary Information.

### Relationship between the transients of O K-edge and the Ti L-edge

**Fig. 7:**
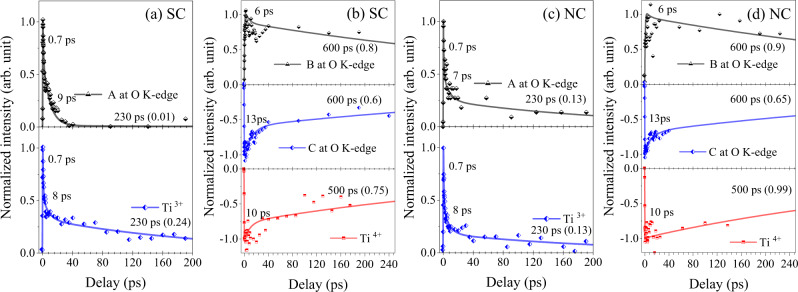
The comparison of the time profiles between major features at the oxygen K-edge and the Ti L-edge. The comparison between the kinetic trace of (**a**) A at the oxygen K-edge and Ti^3+^ at the Ti L-edge for SC, (**b**) B and C at the oxygen K-edge and Ti^4+^ at the Ti L-edge for SC, (**c**) A at the oxygen K-edge and Ti^3+^ at the Ti L-edge for NC, (**d**) B and C at the oxygen K-edge and Ti^4+^ at the Ti L-edge for NC.

Figure 7 shows the comparison of the time profiles between major features at the O K-edge and the Ti L_2,3_-edge. The dynamics of A at the O K-edge shows similar behavior to that of shift-induced transients in the Ti L-edge for both of SC and NC. The fast decay time constants of A (0.7 and 7~9 ps) are similar to those of shift-induced Ti L-edge transients (0.7 and 8 ps). Only the amplitude of the long life time component (230 ps) is different. The dynamics of B and C which are considered as overall shift at oxygen K-edge shows similar behavior to that of 4+ decrease. The decay time constants of C are 0.7, 13, and 600 ps, and those of 4+ decrease are 0.7, 10, and 500 ps which look almost same. For all transients shown in Fig. 7, we can discriminate two different behaviors which are respectively fast (0.7 and 10 ps) and slow (>200 ps). The type of material had little effect on the decay behaviors of holes and electrons during the first 0.7~10 ps, but did have an effect at 10~230 ps. For NC, a long-lifetime (230 ps) component of the hole was observed at A near the VBM at the oxygen K-edge XAS (Fig. 7c). In SC, the long-lifetime component of hole is not clearly seen; if the component exists, it is very small (Fig. 7a). The long-lifetime component (230 ps) of hole appears only near VBM (indicated as A) of NC. It is not seen in SC and other energy region of NC (Fig. 5f). This occurrence of the long lifetime component only in NC suggests that it can be a result of surface-sensitive species. We attribute this surface-sensitive species to the increased pDOS near the VBM in NC, compared to that of bulk^39^. This behavior of surface holes can be exploited by surface-adsorbed species such as hydroxy group (-OH) to trap holes^7,16–18,38^.

In contrast, electrons do not show the surface dependence that the holes show. Excited electrons (as shown as M in Fig. 6) had a long-lifetime component (230 ps) regardless of the material’s size. This result is consistent with previous reports that photo-excited electrons exist both in the bulk and on the surface^16,42^.

## Discussion

Photo-excited carriers form bound excitons followed by harmonic decay (indirect radiative or Auger recombination) or are separated and decay independently (SRH process)^13^. The former is seen as relatively fast decay (on 10 s ps time scale) and the latter as longer-lifetime components (230 ps, or >500 ps) in the transient signals in case of our study (Fig. 7). The faster components represent the initial behavior of the photo-excited carriers including hot-carrier cooling (<0.12 ps)^11–14^, recombination of short-living excitons, and carrier transfer to trap sites^13^. The slower decay can be assigned to free carriers (Drude carriers), remnant results of the geometrical modifications, or indirect effects by trapped carriers. The type of material (SC or NC) had no effect on the slower components in shift-induced Ti L-edge transients, but did affect those of the holes at A. These results suggest that the behavior of excess electrons is not surface sensitive, whereas the behavior of holes is surface sensitive; i.e., the sites of holes with lifetime >230 ps are located near the surface and the position of the energy is near VBM. This difference in behavior is consistent with previous reports^2,16,42^, and supports the validity of this approach to study carrier dynamics. Furthermore, the results of this study suggest simultaneous energy and time-resolved hole dynamics, which have previously been impossible to observe in condensed matter such as metal oxides. For example, in case of electron dynamics, the only observable transients that govern the initial formation of excitations are those of shift-induced Ti L-edge transients. The shortest discernable lifetime component is 0.7 ps. In this time scale, several processes are mixed; hot-electron cooling^13^, recombination^14^, and polaron formation^20–22^. Whereas in case of holes, owing to the energy-level resolving ability, the detailed processes can be discerned: hot-hole cooling (<0.12 ps), hole transfer (0.2 ps), and the resulting gap hole formation <0.32 ps. From the observations (Figs. 2–7), the following mechanism can explain the observed behaviors of photo-excited carriers (Table 1).

**Table 1:** Carrier time scales (comparison between current and reported experiments).

	Electron		Hole	
	References	This work	References	This work
Hot carrier cooling	0.09 ps^11–14^	<0.12 ps	<0.1 ps^2^	<0.12 ps
Exciton recombination	3–13 ps @RT for Si^13^ <<100 ps @ RT @ > 1 mJ/cm^[2 46^	0.7 and 8 ps	3–13 ps @RT for Si^13^ <<100 ps @ RT @ > 1mJ/cm^[2 46^	0.7~10 ps
Polaron (trap state) formation	<0.3 ps^21,22^	<0.7 ps	0.2 ps^16,44^	<0.32 ps
Polaron decay	310 ps, Ti^[3+ 20^	<230 ps	7 us @ 7 K, N. A. @RT^17,18^	8 ps
Defect site decay	500 ps^16^	>500 ps	–	–

**Fig. 8:**
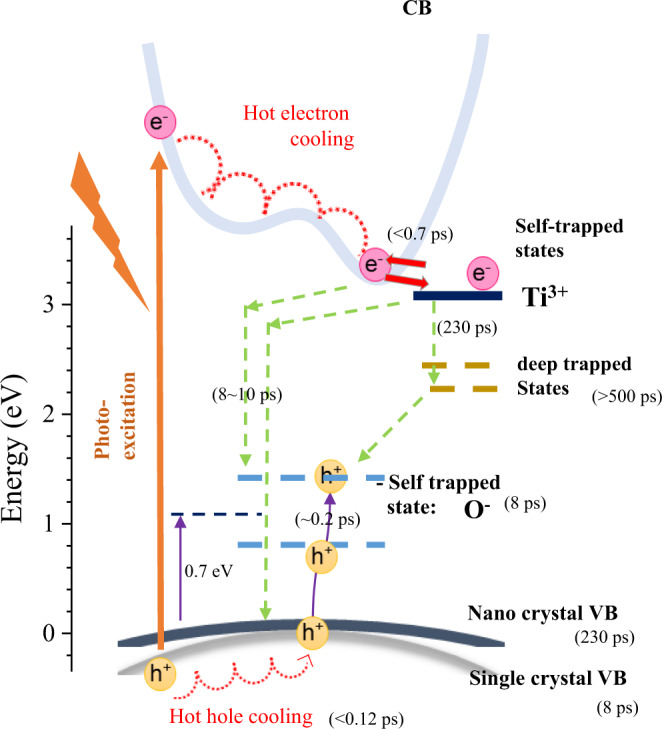
Schematic diagram of the excited carrier dynamics at room temperature. The pump laser excites an electron from VB to CB and thereby generates an e-h pair. Holes deeper than VBM transfer to VBM. Holes at VBM either transfer to gap states or stay in VB. One of the possible gap states can be a self-trapped hole state (Ti-O^−^)^6,9^. Excited electrons are seen in XAS as fast-increasing features followed by fast-decaying features in between peaks (Ti^3+^) of the ground state XAS spectrum. These fast features reflect transient localization of the electron cloud to Ti atoms (Ti^3+^-O)^4^. Most of the localized electrons (80%) recombine with holes very rapidly and only a portion of the electrons (20%) remain. The fast-decrease followed by very slow-decay features at peak positions (Ti^4+^) of the ground XAS spectrum represent the CB electron, effects resulting from geometry relaxations induced by the fast transient electrons, or electrons in deep trap sites.

The process of the holes and electrons can be described as follows (Fig. 8):

### In the first 0.7 ps after the pump laser strikes, three processes are possible

(i) Hot-carrier cooling^13,14,43^ and exciton recombination^13,14^.

(ii) Electron polaron formation (Ti^3+^/O^2−^): except for the time difference in agreement with previous reports that electron polaron formation time is 0.3 ps^21,22^.

(iii) Gap hole formation: some proportion of holes transfers to gap states. Hot hole cooling occurs faster than 0.12 ps within VB and hole transfer to gap states occurs in 0.2 ps from VBM. Therefore, the total time to form trapped hole is <0.32 ps, which is similar to previous reports^44^. The gap holes can be assigned to hole polarons (Ti^4+^/O^1−^). Or the gap holes can be also assigned to Ti_int_ or Vo induced donor-like stats^9,38^.

1. From 0.7 to 10 ps, electrons (in CB or polaronic Ti^3+^) and holes (in VB or gap states) recombine harmonically as indirect phonon assisted radiative recombination or as Auger recombination^14,45^.
2. From 10 to 230 ps, electrons in polaronic Ti^3+^ or CB relax. This is related to previous reports of 100~300 ps electron lifetime at high fluence excitation^20,46^. Or the electrons may fall into bulk deep trap sites^15,16^; if so, electrons in polaronic Ti^3+^ or CB decay to the deep trap sites in 230 ps. (Fig. 8) (If this hypothesis is right, the deep trap sites should not possess Ti 3d character, since we could not observe shift-induced Ti transients longer than 230 ps.)
3. [Only in NC]: From 10 to 230 ps, valence holes in the surface relax. This tendency of the hole is also explained in previous reports^2,16,44^. We currently attribute this to the increased pDOS near the VB edge in NC, compared to that of bulk^39^.
4. After 500 ps, the electrons that are trapped in deep bulk trap sites recombine through SRH pathway, where they remain for >500 ps, which is consistent with previous report. (Fig. 8)^16^ Or this decay can be attributed to modified geometry relaxation.

Referring to the previous reports using hard X-ray pump-probe experiments, both in the bulk and on the surface of anatase TiO_2_ NC in a liquid environment, electron localization occurs in 90 fs and is followed by a structural change that completes the formation of a polaron in 0.3 ps^22^. This process also explains our observed dynamics (<0.7 ps and <0.32 ps for electron and hole polaron formation, respectively), even though ours were obtained using a different absorption energy range and a different sample environment than the previous observations. The decay transients of bare anatase TiO_2_ NC measured by XAS at the Ti K-edge (hard X-ray) could be fit to a bi-exponential function with decay time constants of 310 ps and 6 ns^20^. This result is also consistent with our measurement from the Ti L-edges and oxygen K-edge, which have decay times of ~230 ps and >500 ps. The origin of the 230 ps lifetime is the same as given in ref. ^20^. We may assign the >500 ps life time to deep defect sites, which may be also equivalent to that of 6 ns in ref. ^20^ or to geometry modification effect which will be discussed below.

The question on the interpretation of features B-E (>600 ps) and 4+ decrease (>500 ps) in relation with shift-induce Ti L-edge transients (230 ps) should be discussed. EPR is slower than electron hopping, so it can see a difference between a localized and delocalized state. In contrast, XAS is fast process (<1 fs) so it always sees a 3d electron that is sitting on a specific 3d atom; therefore, XAS can (in first approximation) not distinguish between localized and delocalized states, so electrons on polaronic Ti^3+^ states and CB will both be visible as shift-induced Ti L-edge transient. The experimental transients of 4+ decrease in the Ti L-edge and B-E in the oxygen K-edge XAS are similar since they showed immediate change at excitation and stayed with long life time >500 ps (Fig. 7). These observations mean that the system does not recover to the original states in 500 ps. We can interpret these slow transients as^1^ free conduction electrons which do not participate in recombination with holes, and that have a lifetime >500 ps, or^2^ geometry modification effects followed by quick electron movement or^3^ indirect effects of electrons which are trapped in deep defect sites (where the defect sites should not have Ti 3d character)^16^. The shift-induced Ti L-edge transient have relatively short life time of ~230 ps. Therefore, the electrons in the CB should have lifetimes of at most 230 ps. This reasoning exclude the possibility of a CB origin of the >500 ps life time. We currently assign the >500 ps lifetime to the geometry modification effects or to indirect effects by deep trap sites^16^. We also assign the 230 ps lifetime either to free electrons in the CB or to localized electrons in polaronic Ti^3+^. The above two possibilities are not discernable currently. Therefore, we expect that future study can elucidate this inscrutable phenomena.

Finally, we would like to discuss the physical meaning of the shift-induced and the other effects in the transient spectra at the Ti L-edge. The minor component at 1 ps and the major components at 100 ps look similar in character since they are almost unchanged whereas the major component at 1 ps significantly decreased at 100 ps (Supplementary Fig. S9). The major component at 1 ps can be assigned as a rigid shift of the whole spectrum. Whereas the assignment of the major component at 100 ps is not straightforward because the simulations do not fit perfectly by any method. We have used several models that can best explain the major component at 100 ps as shown in Supplementary Fig. S9. Using two models, we can approximately simulate the peculiar transient spectrum at 100 ps which shows negative values at peak positions of the GS spectrum and shows almost zero intensity for positive values. One method uses the second derivative of the GS and the other method uses a broadening of the GS. Firstly, if we twice differentiate the GS, the peak positions of the GS show negative values in which the amplitude is larger as the peak is sharper (Supplementary Fig. S9a). Secondly, if we assume the excited spectrum as broadening of the GS spectrum according to Supplementary Eq. (S5), similar effects to that of second derivative are obtained but the sensitivity of the sharpness of each peak is moderated. We notice that both the effects by second derivative and broadening of GS can partially explain the peculiar transients which are not the shift-induced component. These two effects are currently assumed to be originated from geometrical modification or from indirect effects by trapped electrons in unidentified trap sites.

## Methods

### X-ray spectroscopy measurements

Electron-yield Tr-XAS spectra were obtained at the SSS beamline at the Pohang Accelerator Laboratory X-ray Free Electron Laser (PAL-XFEL), using a femtosecond laser and a data-acquisition setup^47^. The X-rays were focused to a spot size of 30 µm × 30 µm by Kirkpatrick–Baez mirrors, and the laser spot size was 100 µm (all values FWHM). The samples were excited with 100 fs pulses at 266 nm (4.66 eV) at a fluence of 20 mJ/cm^2^. The electron yield was measured using a microchannel plate at normal incidence to the X-ray beam. The sample was changed regularly to avoid problems with aggregation and any possible long-term sample changes.

### Calculations

#### Quantum ESPRESSO

The electronic structure within the DFT was obtained using the Quantum ESPRESSO package^32,33^. The theoretical calculations were performed using a plane-wave basis set and norm-conserving pseudopotential in a Fritz-Haber-Institute scheme. We used the Trouiller Martins type PW-LDA exchange-correlation functional for oxygen 2s^2^ and titanium 4s^2^ 3d^2^ 3p^6^ (valence electrons). The Brillouin zone summations were conducted over a 2 × 2 × 2 k-points grid for a unit cell containing 6(Ti_2_O_4_) atoms. Electronic smearing with a width of 0.002 Ry was applied according to the Gaussian method. The plane wave energy and charge density cut-offs were 40 Ry and 140 Ry, respectively, corresponding to a calculation accuracy of 0.7 mRy per atom.

#### OCEAN

The oxygen K-edge spectra simulations of TiO_2_ unit cell were performed using the OCEAN package^30,31^ that implements the Bethe-Salpeter equation (BSE) approximation, which is built upon the DFT ground-state charge density and Kohn–Sham Hamiltonian. The DFT routine was performed with the Quantum ESPRESSO package^33^. Local density approximation (LDA) was used for the exchange-correlation functional. Norm-conserving pseudopotentials from the ABINIT distribution were used, in conjunction with a cutoff energy of 140 Ry. The size of the k-point grid used to solve the Kohn–Sham states for BSE was 4 × 4 × 4, and the screening calculations for both structures used a 2 × 2 × 2 k-point grid. The number of unoccupied bands used for the BSE calculation was at least 50, and the screened core-hole potential calculation included at least 100 bands. Each oxygen atom in the simulation cell was considered as the absorbing atom. The polarization vectors were set to be [100], [010], and [001], and the final spectrum was obtained by averaging the spectra generated by all oxygen atoms, by using each of the polarization vectors.

### Reporting summary

Further information on research design is available in the Nature Research Reporting Summary linked to this article.

## Supplementary information

Supplement Information

Reporting Summary

Lasing Reporting Summary

## Data Availability

The data generated in this study are provided in the Supplementary Information/Source Data file. Source data are provided with this paper.

## Source data

Source Data
