# Supplementary material for: Direct and real-time observation of hole transport dynamics in anatase TiO2 using X-ray free-electron laser
Source: Nat Commun. 2022 May 9;13:2531. doi: 10.1038/s41467-022-30336-1 (PMC9085873; doi:10.1038/s41467-022-30336-1)
Supplement: Supplementary file 1 — Supplement Information [file 41467_2022_30336_MOESM1_ESM.pdf]

Supplementary Materials:

Direct and real-time observation of hole transport dynamics in anatase TiO_2 using X-ray free-electron laser

Sang Han Park[1], Abhishek Katoch[1], Keun Hwa Chae[2], Sanjeev Gautam[3], Piter Miedema[4], Sang Wan Cho[5], Minseok Kim[1], Ru-Pan Wang[6], Masoud Lazemi[6], Frank de Groot[6*], and Soonnam Kwon[1*]

[1]Pohang Accelerator Laboratory, Pohang, Gyeongbuk 37673, South Korea

[2]Korea Institute of Science and Technology, Seoul, South Korea

[3]Dr S. S. Bhatnagar University Institute of Chemical Engineering & Technology, Panjab University, Chandigarh 160014, India

[4] European XFEL Gmbh, Notkestrasse 85, D-22607 Hamburg, Germany

[5]Department of Physics, Yonsei Univerity, Wonju, South Korea

[6] Materials Chemistry and Catalysis (MCC), Debye Institute for Nanomaterials Science, Utrecht University, Universiteitslaan 99, 3584 CG Utrecht, The Netherlands

*Correspondence to: f.m.f.degroot@uu.nl (F. de Groot) and snkwon@postech.ac.kr (S. Kwon)

Sample preparation

TiO_2 nanoparticles (< 22 nm) were purchased from Sigma-Aldrich (798495); they were pelletized for the measurement. An anatase TiO_2 (001) single crystal was purchased from MTI Corp., USA.

Time-resolved X-ray absorption spectroscopy

All XAS measurements were performed at the SSS beamline at the PAL-XFEL (Pohang, Korea). The X-rays were monochromatized by a grating monochromator that has an energy resolution of ≤ 0.2 eV. The spectra were recorded in electron yield mode (EY) and fluorescence yield (FY) mode using a multi-channel plate detector. The signal from the sample was normalized to the incident X-ray flux monitored using an MCP with a central hole. The femtosecond time-resolved XAS was conducted using a laser with a 100 fs pulse width at 266 nm and an XFEL with a 80 fs pulse width. The X-ray pulse probed the sample at adjustable delay time (excited state) and reference delay time (-15 ps, ground state) alternately to compensate for the fluctuation of the experimental condition: i.e., when we measured the XAS signal by considering time delay from 0 to 2 ps, we measured for 5 s each in the order 0, -15, 1, -15, 2, and -15 ps, and repeated several times for the whole process. The transient signal was calculated by subtracting the signal from the subsequent reference signal. The X-ray beam was focused to a 30 μm spot (FWHM) on the sample by a pair of Kirkpatrick-Baez mirrors, to ensure overlap with the laser focus.

The delay scans were measured as long as ~250 ps for the major features A, B, C, Ti^{3+} and Ti^{4+} except feature B of the nanocrystal. (Fig. 5c and Fig. 7) The other delay scans were measured as long as 40 ps due to limited beamtime. (Fig. 4 and Fig. 5 a, b, d, e) Each delay scan was averaged 5 to 10 times depending on the signal level.

Optical laser pump

A Ti:sapphire laser pulse with a duration of 100 fs and a wavelength of 266 nm (4.66 eV) was focused to 100 μm in FWHM. The fluence of the pump laser was 20 mJ/cm^2. No significant change was not observed in the transient features and life time behaviors in the fluence range of 10 ~ 30 mJ/cm^2. Laser fluence up to 50 mJ/cm^2 did not damage the sample. A laser fluence below 10 mJ/cm^2 induced transient signals that were too weak to obtain a sufficient signal-to-noise ratio.

The concentration of excited electrons could be calculated using following parameters to predict the fraction of the charge reduction of the Ti atoms. The number of atoms in a unit cell of anatase TiO_2 is 12 (Ti_4 O_8) and the average fluence of the pump laser is 20 mJ/cm^2. Assuming the absorption depth of laser as 100 nm and the atomic density of TiO_2 is 3 x 10^{28} /m^3, the photon number per atom is calculated as 0.09, and therefore the electron numbers per unit cell is 1.08. At this high concentration of free electrons (Drude electron), atoms from the regular lattice will be reduced. This value is also comparable with the intensity ratio between the transient spectrum and the ground spectrum (0.1 for Ti L-edge). Therefore, the spectral shift is caused by oxidation state change from Ti^{4+} to Ti^{3+} (O^{2-} to O^{1-}) for the atom where an electron is excited. The number of excited electrons and the change in spectral intensity can be compared in soft X-ray energy range because the escape depth of electrons (~few nm) is shorter than the absorption depth of the laser (~few tens of nm).

Fitting kinetic traces of the X-ray signals

Most of the kinetic transients in the manuscript were fitted using a tri-exponential fit function with a Heaviside function (S-1) or bi-exponential fit function with exponential growth function (S-2).

Most of the kinetic transients were fitted using the following function.

$$I(\Delta\mathbf{t}) = \text{heaviside}(\Delta \text{t}) \times \left\{ \frac{I_1}{I_1+I_2+I_3} e^{\frac{\Delta t}{\tau_1}} + \frac{I_2}{I_1+I_2+I_3} e^{\frac{\Delta t}{\tau_2}} + \frac{I_3}{I_1+I_2+I_3} e^{\frac{\Delta t}{\tau_3}} \right\}, \quad \text{(S-1)}$$

where the $heaviside(\Delta t)$ is the Heaviside function convoluted with a Gaussian function to account for the finite time resolution, and I_1, I_2, I_3, τ_1, τ_2, and τ_3 are the amplitude and the relaxation time constant Table S1). (*S1*) The width of the Gaussian function was 0.12±0.01 ps.

For the slow-rising transients (such as "B" in Fig. 4) the following function was used for curve fitting.

$$I(\Delta \text{t}) = \left(1 - e^{\frac{-\Delta t}{\tau_{rise}}}\right) \times \left(\frac{I_1}{I_1+I_2} e^{\frac{\Delta t}{\tau_1}} + \frac{I_2}{I_1+I_2} e^{\frac{\Delta t}{\tau_2}}\right), \quad \text{(S-2)}$$

where the τ_{rise} is the growth time constant, I_1, I_2, τ_1, and τ_2 are the amplitude and the relaxation time constant (Table S1). (*S2*)

<u>Formula used in the simulation of transient spectra (Fig. S8 and S9)</u>

The transient spectra are supposed to originate from a rigid shift of ground state spectrum (GS(x)) due to the chemical shift and spectral broadening. The shifts can be expressed as (S-3) and the broadening effect as (S-4).

$$\Delta XAS = S_{shift}[C_1 \; GS(x - \Delta E) - GS(x)] \quad \text{(S-3)}$$

$$\Delta XAS = S_{brdn}\left[C_2 \; \frac{GS(x-\Delta)+GS(x+\Delta)}{2} - GS(x)\right] \quad \text{(S-4)}$$

$$\Delta XAS = S_{2ndD}\left[\; \frac{d^2}{dx^2} GS(x)\right] \quad \text{(S-5)}$$

Where S_{shift} (or S_{brdn}, S_{2ndD}) is weighting parameter, C_1 (or C_2) is attenuation parameter, ΔE is the amount of rigid energy shift, and Δ is the quantity of broadening.

We used the method as given for the Ti K-edge in ref 20. The result is summarized in Eq. S3-5 and Fig. S9. The transient spectrum at each delay time consists of two components: a rigid shift (-0.6 eV) due to localization of electrons and effects which are not originated from the localization. The former dominates at 1 ps whereas the later dominates at 100 ps. In other words, the former disappears relatively fast whereas the later maintains its influence until 100 ps. The origin of the later component is not identified yet. We simulated it using various mathematical treatments. As shown in Fig. S9, broadening of the spectrum (resulting in a reduction of peak intensity) may be one of several causes. Firstly, the broadening can be caused by electron transfer from a localized Ti^{3+} site to relatively delocalized defect states, or by geometric modification caused by electrons in Ti^{3+} or in the defect states (S7). Secondly, the second derivative of the spectrum can also explain the long surviving component qualitatively. Even though we could not explain its exact physical meaning, it resembles the broadening effect. (Note that at the maxima, the 2nd derivative shows minus value and at minima it shows positive value. If peaks are sharper, the absolute of the minus value shows higher value)

Table S1. Fitting parameters used in Figs. 4 - 7.

		Crystal	Decay 1	Decay 2	Decay 3	
O K -edge	“A”	Single	0.7 (0.5)*	9 (0.49)	230 (0.01)	EY**
		Nano	0.7 (0.6)	8 (0.2)	230 (0.2)	FY**
		Nano	0.7 (0.6)	7 (0.27)	230 (0.13)	EY
	Gap (VBM +1.1)	Single	0.7 (0.79)	8 (0.2)	230 (0.01)	EY
		nano	0.7(0.5)	8 (0.45)	230 (0.02)	EY
	“B”		growth	Decay 1	Decay 2	
		Single	-	6 (0.2)	600*** (0.8)	EY
		Nano	-	6 (0.1)	600*** (0.9)	FY
	“C”		Decay 1	Decay 2	Decay 3	
		Single	0.7 (0)	13 (0.4)	600*** (0.6)	EY
		Nano	0.7 (0.1)	13 (0.25)	600*** (0.65)	EY
Ti L -edge	Ti^{3+}	Single	0.7 (0.6)	8 (0.16)	230 (0.24)	EY
		Nano	0.7 (0.69)	8 (0.18)	230 (0.13)	EY
	Ti^{4+}	Single	0	10(0.25)	500*** (0.75)	EY
		nano	0	10 (0.01)	500*** (0.99)	EY

*Life times are measured in pico seconds. Values in parenthesis indicate the amplitude of the lifetime components.

**EY: electron yield; FY: fluorescence yield., respectively.

***Measurements were conducted as long as 250 ps. The longer life times were obtained from extrapolation of the data.

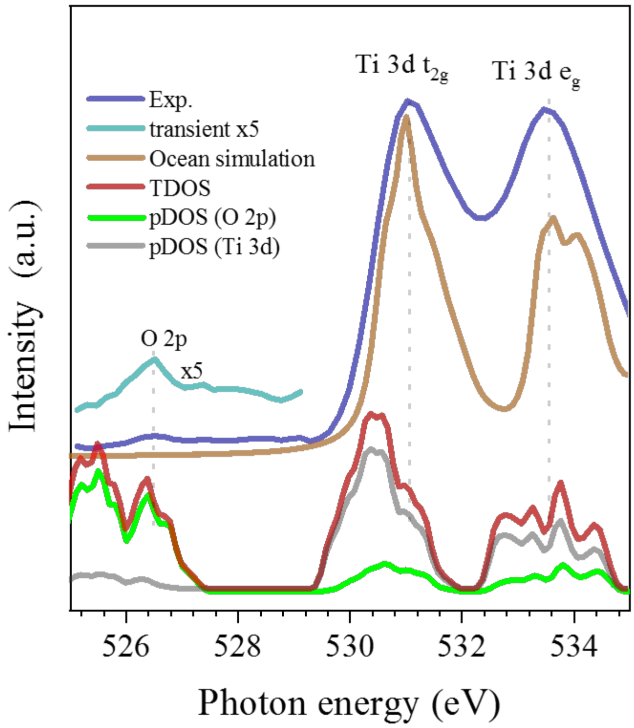

Fig. S1. O K-edge XAS Spectra and comparison with calculation for TiO_2. DFT calculations were performed using Quantum ESPRESSO (S3, S4) and the simulation of the spectra was performed using the Ocean software (S5, S6).

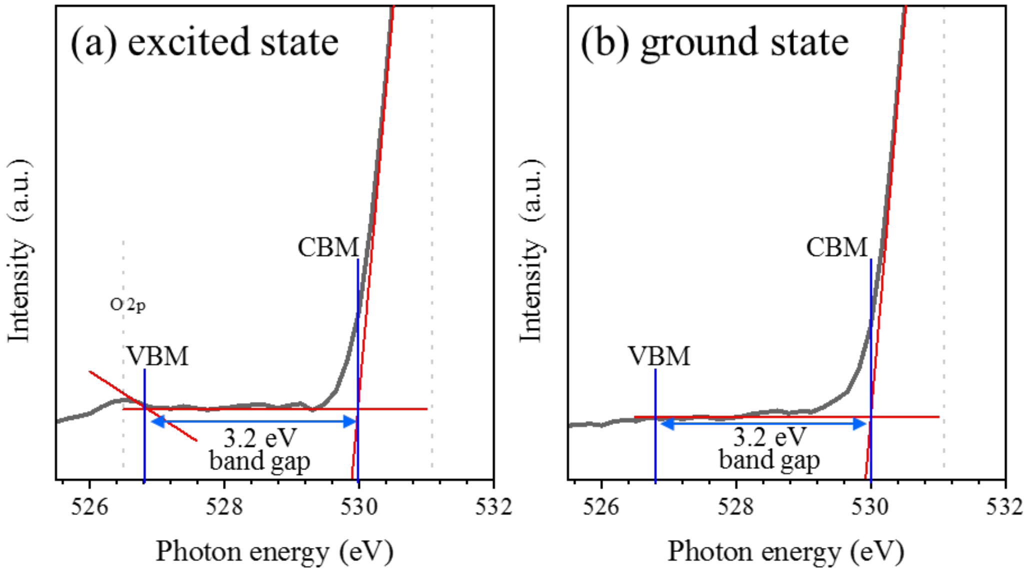

Fig. S2. Determination of the band gap using XAS at the O K-edge by the linear extrapolation method for the excited state (a) and the ground state (b). The VBM of the ground state was determined by subtracting 3.2 eV of the known band gap from the CBM.

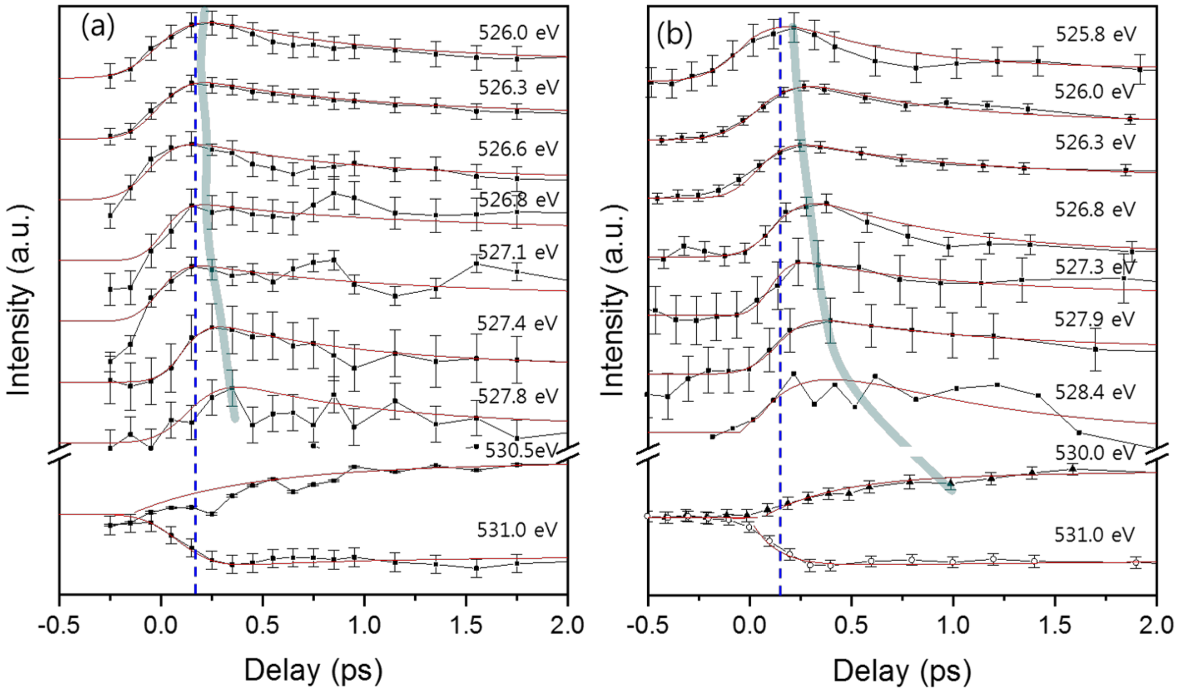

Fig. S3. Normalized data of transient signals shown in Fig. 4 for (a) single crystal and (b) nanocrystal.

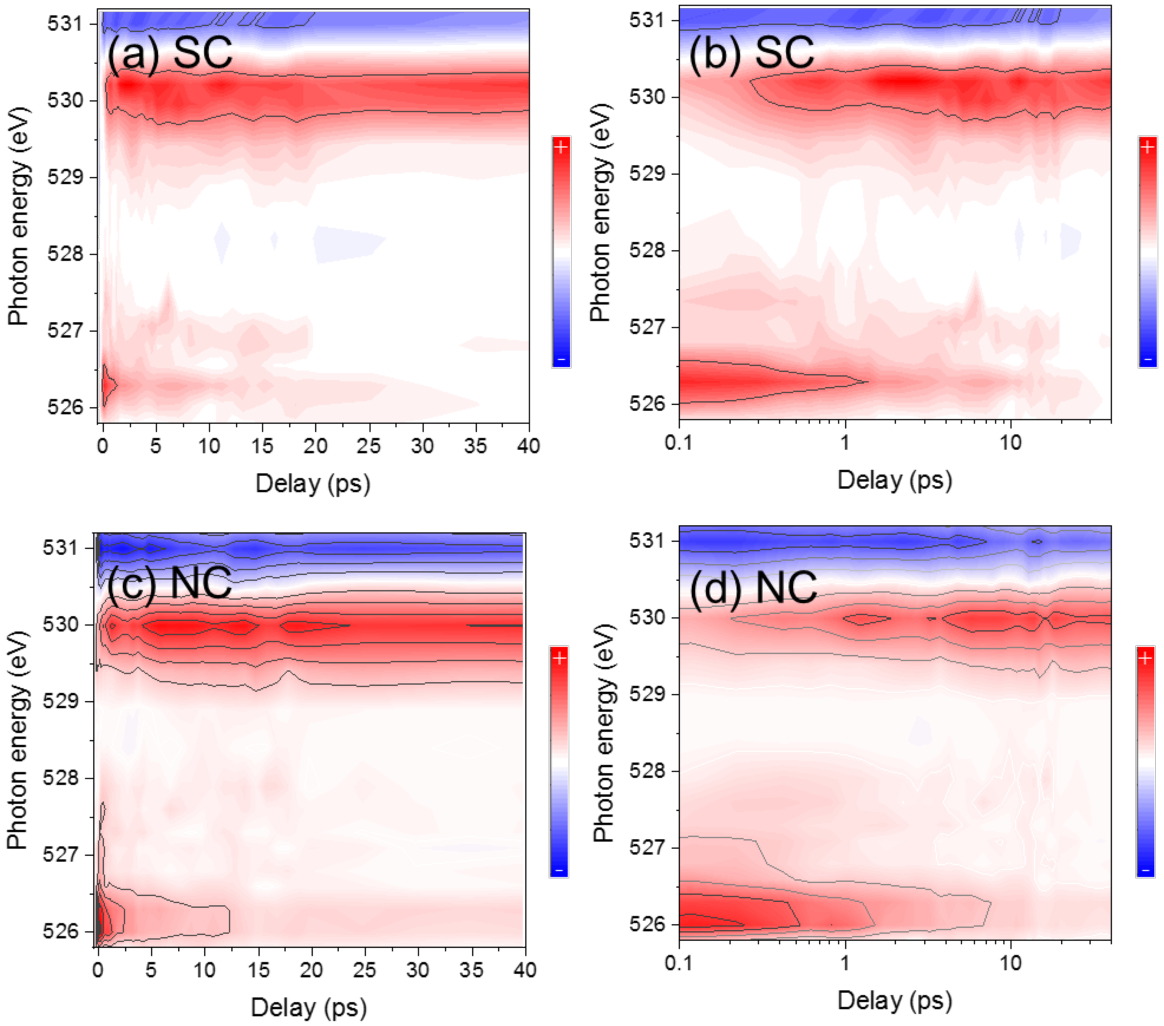

Fig. S4. Experimental data of transient signal as a function of pump-probe delay up to 40 ps at the O K-edge in Fig. 4 of the manuscript. (a and b) show the data for anatase TiO_2 single crystal in linear (a) and log (b) scale. (c and d) show the data for anatase TiO_2 nanocrystal in linear (c) and log (d) scale.

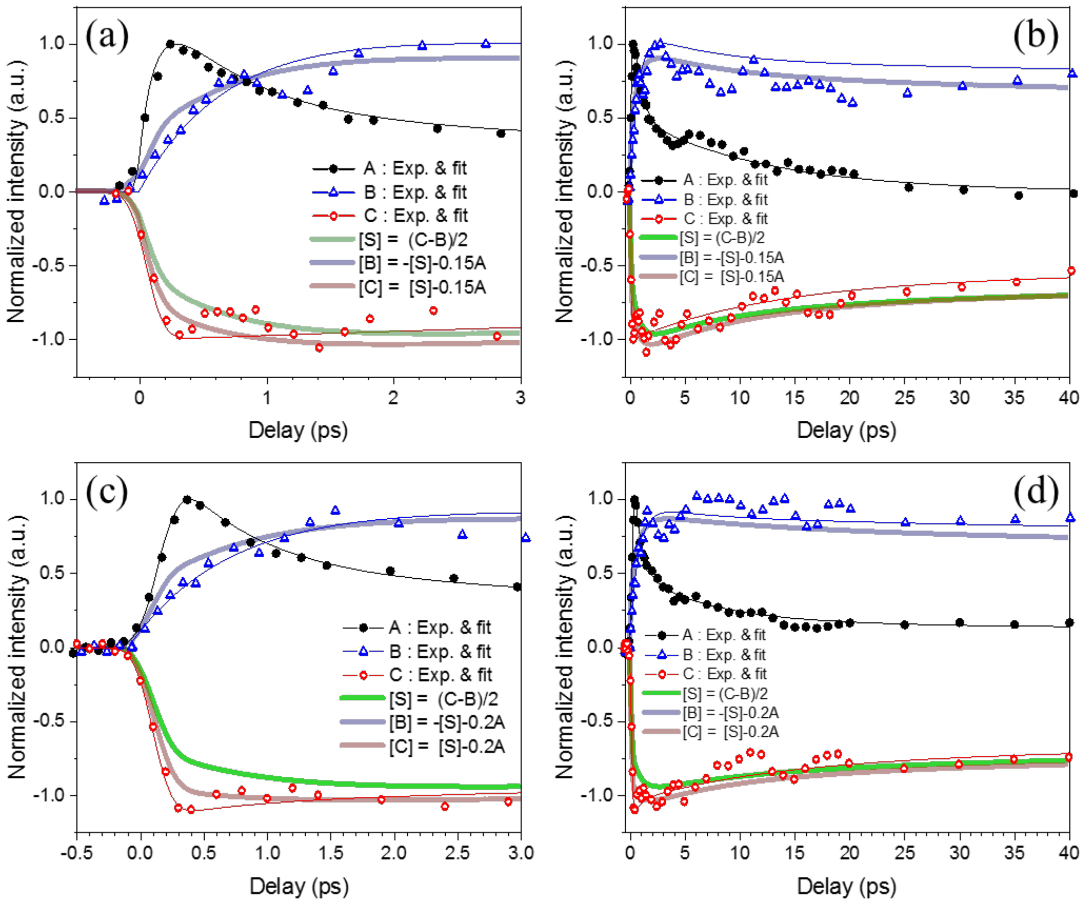

Fig. S5. Comparison of time profiles of experimental and model calculations at O K-edge in Fig. 4 of the manuscript. (a and b) show the comparion at initial (~ 3 ps) and long (~ 40 ps) time delays for single crystal. (c and d) show the comparion at initial (~ 3 ps) and long (~ 40 ps) delay time range for nanocrystal.

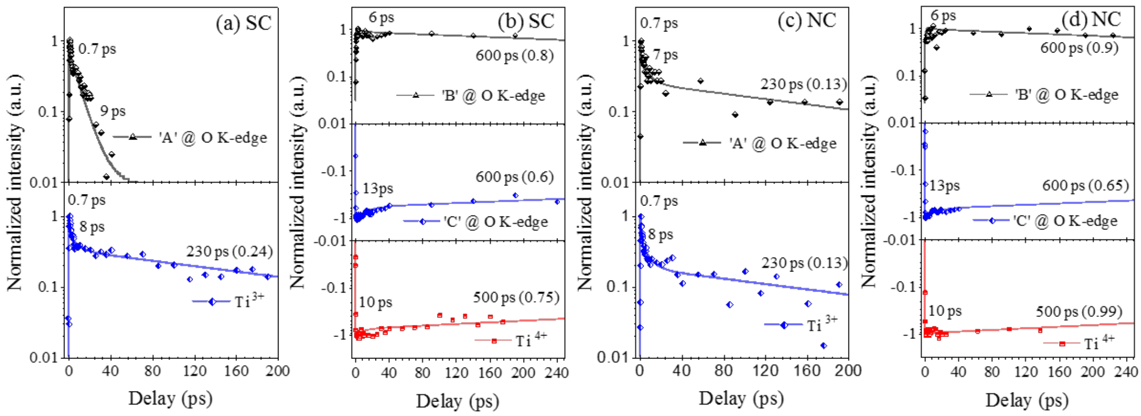

Fig. S6. The comparison of the time profile between major features at the O K-edge and the Ti L-edge in intensity versus log scale using the same data in Fig. 7 in the manuscript. The comparison between the kinetic trace of (a) 'A' at O K-edge and Ti^{3+} at Ti L-edge for a single crystal, (b) 'B' and 'C' at O K-edge and Ti^{4+} at Ti L-edge for a single crystal, (c) 'A' at O K-edge and Ti^{3+} at Ti L-edge for nanocrystal, (d) 'B' and 'C' at O K-edge and Ti^{4+} at Ti L-edge for the nanocrystal.

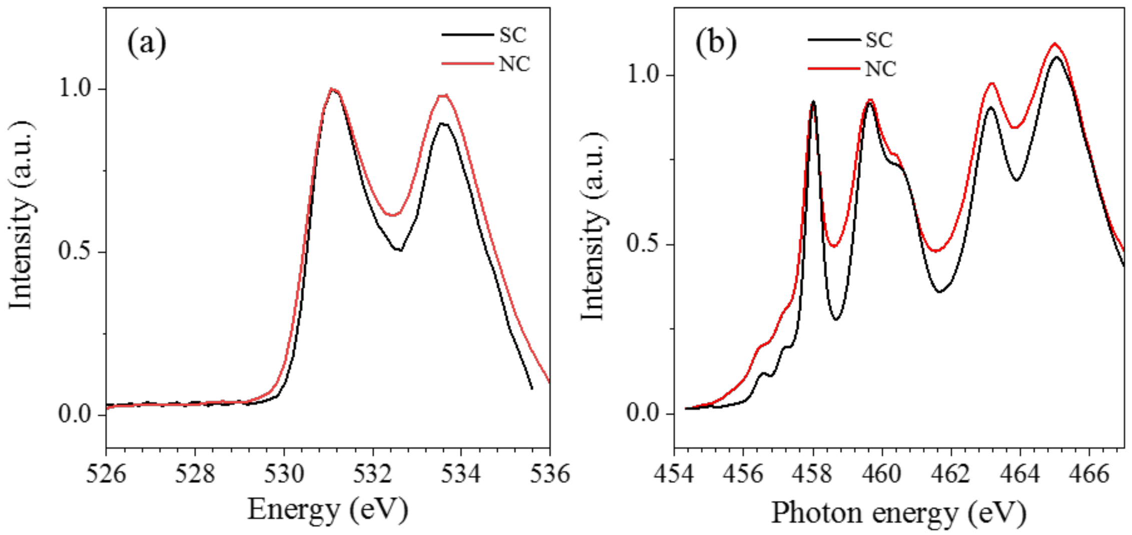

Fig. S7. The comparison of the ground state spectra between SC and NC for the O K-edge (a) and Ti L-edge (b). The ground state XAS spectrum of NC shows broad features compare to that of SC and this represent there is more defects in NC than SC.

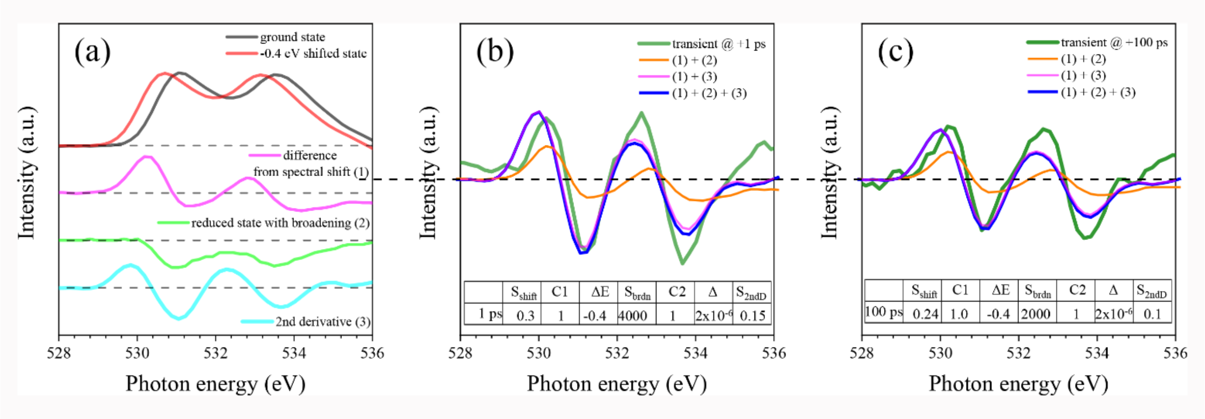

Fig. S8. Simulation of transients at the oxygen K-edge XAS for TiO_2 NC. (a) Comparison between effects of spectral shift and spectral broadening. Comparison between experimental and simulated transient at 1 ps (b) and at 100 ps (c).

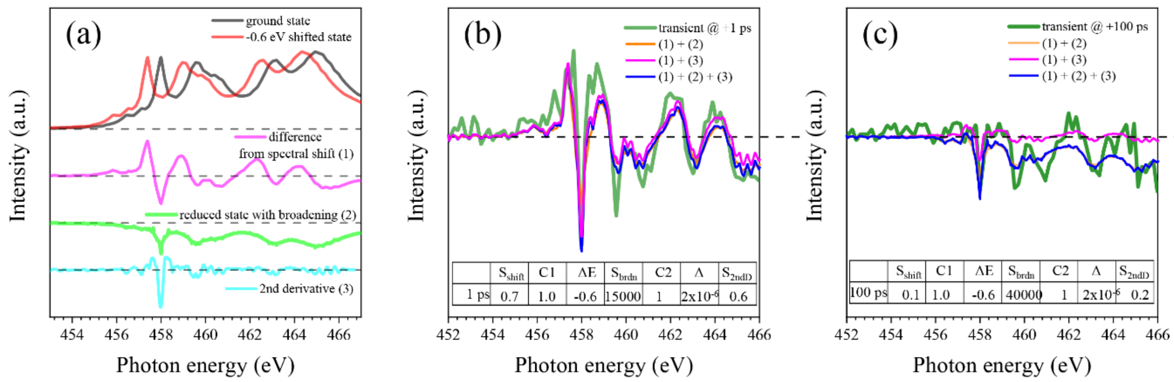

Fig. S9. Simulation of transients at the Ti L-edge XAS for TiO_2 NC. (a) Comparison between effects of spectral shift and spectral broadening. Comparison between experimental and simulated transient at 1 ps (b) and at 100 ps (c).

References.

S1. Varnavski, O. P. *et al.*, Femtosecond excitation dynamics in gold nanospheres and nanorods. *Phys. Rev. B* **72,** 235405 (2005)

S2. Obara, Y. *et al.*, Femtosecond time-resolved X-ray absorption spectroscopy of anatase TiO_2 nanoparticles using XFEL. *Struct. Dyn.* **4,** 044033-1-16 (2017).

S3. Wang, Y.-G. *et al.*, The Role of Reducible Oxide−Metal Cluster Charge Transfer in Catalytic Processes: New Insights on the Catalytic Mechanism of CO Oxidation on Au/TiO_2 from ab Initio Molecular Dynamics. *J. Am. Chem. Soc.* **135,** 10673-10683 (2013).

S4. Morgan, B. J., and Watson, G. W. Polaronic trapping of electrons and holes by native defects in anatase TiO_2. *Phys. Rev. B* **80,** 233102-4 (2009).

S5. Iacomino, A. *et al.*, Structural, electronic, and surface properties of anatase TiO_2 nanocrystals from first principles. *Phys. Rev. B* **78,** 075405 (2008).

S6. Tamaki, Y. *et al.*, Dynamics of efficient electron–hole separation in TiO_2 nanoparticles revealed by femtosecond transient absorption spectroscopy under the weak-excitation condition. *Phys. Chem. Chem. Phys.*, **9**, 1453-1460 (2007).

S7. Thakur, H. *et al.* Modifications in structural and electronic properties of TiO2 thin films using swift heavy ion irradiation *J. Appl.. Phys.* **110,** 083718 (2011).
