# Supplementary material for: Direct and real-time observation of hole transport dynamics in anatase TiO2 using X-ray free-electron laser
Source: Nat Commun. 2022 May 9;13:2531. doi: 10.1038/s41467-022-30336-1 (PMC9085873; doi:10.1038/s41467-022-30336-1)
Supplement: Supplementary file 3 — Lasing Reporting Summary [file 41467_2022_30336_MOESM3_ESM.pdf]

nature research

Corresponding author(s): NCOMMS-21-36192B

Lasing Reporting Summary

Nature Research wishes to improve the reproducibility of the work that we publish. This form is intended for publication with all accepted papers reporting claims of lasing and provides structure for consistency and transparency in reporting. Some list items might not apply to an individual manuscript, but all fields must be completed for clarity.

For further information on Nature Research policies, including our data availability policy, see Authors & Referees.

Experimental design

Please check: are the following details reported in the manuscript?

1. Threshold

Plots of device output power versus pump power over a wide range of values indicating a clear threshold

☐ Yes
☒ No

Our experiments are not related to the device emitting laser.

2. Linewidth narrowing

Plots of spectral power density for the emission at pump powers below, around, and above the lasing threshold, indicating a clear linewidth narrowing at threshold

☐ Yes
☒ No

Our experiments are not related to the emission of laser.

Resolution of the spectrometer used to make spectral measurements

☒ Yes
☐ No

The X-rays were monochromatized by a grating monochromator that has an energy resolution of ≤ 0.2 eV. in Supplementary Information.

3. Coherent emission

Measurements of the coherence and/or polarization of the emission

☐ Yes
☒ No

Our experiment do not require the coherence or polarization dependence.

4. Beam spatial profile

Image and/or measurement of the spatial shape and profile of the emission, showing a well-defined beam above threshold

☐ Yes
☒ No

This experiment do not require any spatial profile of pump laser.
In case of our optical pump-XFEL probe experiment, the X-rays were focused to a spot size of 30 µm x 30 µm by Kirkpatrick-Baez mirrors, and the pump laser was focused to a spot size of 100 um x 100 µm with Gaussian profile (all values in FWHM).

5. Operating conditions

Description of the laser and pumping conditions
Continuous-wave, pulsed, temperature of operation

☒ Yes
☐ No

"The samples were excited with 100 fs pulses at 266 nm (4.66 eV) at a fluence of 20 mJ/cm2." in Method section.

Threshold values provided as density values (e.g. W cm^{-2} or J cm^{-2}) taking into account the area of the device

☒ Yes
☐ No

"Laser fluence < 10 mJ/cm2 induced transient signals that were too weak to obtain a sufficient signal-to-noise ratio." in Supplementary Information.

6. Alternative explanations

Reasoning as to why alternative explanations have been ruled out as responsible for the emission characteristics
e.g. amplified spontaneous, directional scattering; modification of fluorescence spectrum by the cavity

☒ Yes
☐ No

We do not analyze the emission characteristics but we observe absorption of X-ray. For the interpretation of XAS signal, we fully discussed the possibilities of another explanations in "Discussion" section. One example can be found in the text as follows: "We also assign the 230 ps lifetime either to free electrons in CB or to localized electrons in polaronic Ti3+. The above two possibilities are not discernable currently. " in Discussion section.

7. Theoretical analysis

Theoretical analysis that ensures that the experimental values measured are realistic and reasonable
e.g. laser threshold, linewidth, cavity gain-loss, efficiency

☒ Yes
☐ No

To simulate density of state and x-ray absorption spectra, we performed theoretical calculation based on DFT using Quantum ESPRESSO and OCEAN packages.
"Quantum ESPRESSO
The electronic structure within the DFT was obtained using the Quantum ESPRESSO package. (32, 33) The theoretical calculations were performed using a plane-wave basis set and norm-conserving pseudopotential in a Fritz-Haber-Institute scheme. We used the Trouiller Martins type PW-LDA exchange-correlation functional for oxygen 2s2 and titanium 4s2 3d2 3p6 (valence electrons). The Brillouin zone (BZ) summations were conducted over a 2×2×2 BZ k-points grid for a unit cell containing 6(Ti2O4) atoms. Electronic smearing with a width of 0.002 Ry was applied according to the 'Gaussian' method. The plane wave energy and charge density cut-offs were 40 Ry and 140 Ry, respectively, corresponding to a calculation accuracy of 0.7 mRy per atom.
OCEAN
The oxygen K-edge spectra simulations of TiO2 unit cell were performed using the OCEAN package (30, 31) that implements the Bethe-Salpeter equation (BSE) approximation, which is built upon the DFT ground-state charge density and Kohn-Sham Hamiltonian. The DFT routine was performed with the Quantum ESPRESSO package. (33) Local density approximation (LDA) was used for the exchange-correlation functional. Norm-conserving pseudopotentials from the ABINIT distribution were used, in conjunction with a cutoff energy of 140 Ry. The size of the k-point grid used to solve the KohnSham states for BSE was 4×4×4, and the screening calculations for both structures used a 2×2×2 k-point grid. The number of unoccupied bands used for the BSE calculation was at least 50, and the screened core-hole potential calculation included at least 100 bands. Each oxygen atom in the simulation cell was considered as the absorbing atom. The polarization vectors were set to be [100], [010], and [001], and the final spectrum was obtained by averaging the spectra generated by all oxygen atoms, by using each of the polarization vectors." in Method section.

8. Statistics

Number of devices fabricated and tested

☐ Yes
☒ No

Our experiments are not related to device characteristics.

Statistical analysis of the device performance and lifetime (time to failure)

☐ Yes
☒ No

Our experiments are not related to device characteristics.
